# Incarcerated Uterus Presenting as Short Cervix and Placenta Previa

**DOI:** 10.1155/2018/7373507

**Published:** 2018-09-04

**Authors:** Ali Alhousseini, Salam Zeineddine, Adham Alsamsam, Bernard Gonik, Jacques Abramowicz, Karoline Puder, Homam Saker, Edgar Hernandez-Andrade

**Affiliations:** ^1^Department of Obstetrics and Gynecology, Division of Maternal Fetal Medicine, Wayne State University, Detroit, Michigan, USA; ^2^Department of Physiology, Wayne State University, Detroit, Michigan, USA; ^3^Department of Internal Medicine, Wayne State University, Detroit, Michigan, USA; ^4^Department of Obstetrics and Gynecology, University of Chicago, Chicago, Illinois, USA

## Abstract

**Introduction:**

Incarcerated uterus is a rare complication of pregnancy, usually associated with retroversion.

**Case:**

A 26-year-old woman presents at 19 4/7 weeks for evaluation of a short cervix and placenta previa. On ultrasound scan, the placenta was considered previa and the cervix was not visualized. The cervix was not identified by pelvic examination and the presumptive diagnosis of short cervix was done. The patient was followed up closely and remained asymptomatic. Retrospective analysis of the ultrasound images showed a retroverted uterus with an elongated cervix compressed towards the anterior vaginal wall. At 26 weeks of gestation, ultrasound showed a cervical length of 41 mm and a fundal placenta and the diagnosis of spontaneous correction of an incarcerated uterus was made. The patient had an uncomplicated vaginal delivery at 39 3/7 weeks.

**Comment:**

Identification and close follow-up of incarcerated uterus may potentially help in avoiding serious obstetrical and surgical complications.

## 1. Introduction

Incarcerated gravid uterus is a rare complication of pregnancy where a uterus in retroversion fails to ascend into the abdominal cavity [1]. Spontaneous resolution of retroversion usually occurs by 14 weeks of pregnancy [1]. While incarceration may imply pain or discomfort, the term “incarcerated uterus” has been used to describe uterine entrapment or sacculation that is usually asymptomatic [1, 2]. We are reporting an incarcerated uterus mimicking short cervix and placenta previa with spontaneous correction of the incarceration at 26 weeks of gestation and resolution of the above suspected ultrasound findings.

## 2. Case Report

**Figure 1:**
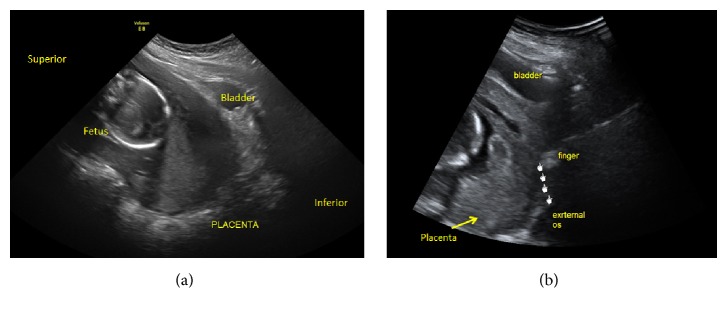
Transabdominal sagittal ultrasound image at 19 weeks showing complete placenta previa (a). Pelvic exam was unable to identify the cervix (b).

**Figure 2:**
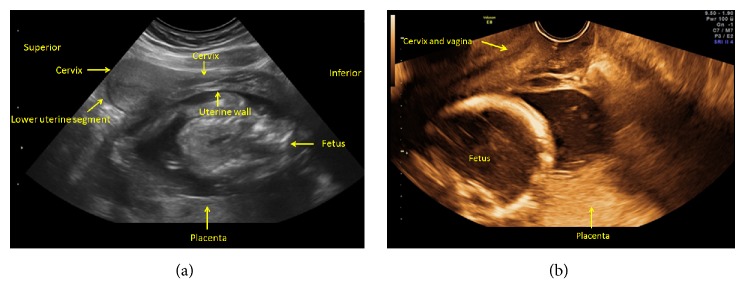
Re-evaluation of the ultrasound images obtained at 19 weeks. Transabdominal (a) and endovaginal (b) ultrasound images showing the anteriorly located and stretched cervix and lower uterine segment.

**Figure 3:**
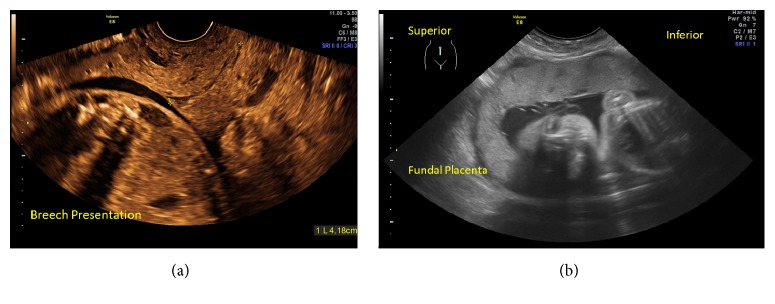
(a) Endovaginal ultrasound at 26 weeks showing a normal cervical length of 4.16 cm. (b) Transabdominal ultrasound showed a fundal placenta with breech presentation.

A 26-year-old woman, Gravida 3, Para 1, Abortus 1, presented at 19 weeks and 4 days of gestation for evaluation of a short cervix and placenta previa. The patient was asymptomatic, denying pain, vaginal bleeding, leakage of fluid, cramping, or uterine contractions. Ultrasound examination suggested a total placenta previa. The cervix was difficult to visualize and was considered unmeasurable in length. The fetus was in a cephalic presentation (Figures 1(a) and 1(b)). During speculum examination, the cervix could not be visualized. Because of the uncertain diagnosis, a careful digital exam was performed to evaluate the location of the external cervical os which was difficult to assess. A transabdominal ultrasound scan was performed concomitant with the digital examination (Figure 1(b)). The diagnosis remained unclear, and therefore expectant management with daily vaginal progesterone therapy was initiated. Weekly transvaginal ultrasound scans continued showing similar findings. The patient remained clinically asymptomatic. Retrospective review of the earlier ultrasound images showed that the cervix was compressed against the anterior vaginal wall. The cervix and the lower uterine segment were elongated and stretched along what was thought to be the anterior wall of the uterus (Figures 2(a) and 2(b)). At 26 weeks of gestation, upon repeat transvaginal ultrasound scanning, the cervix was found to be 41 mm in length and the placenta was in an anterior-fundal position with the fetus in a breech presentation (Figures 3(a) and 3(b)). These new findings supported spontaneous resolution of a retroverted incarcerated uterus. The patient had a normal course of pregnancy afterwards. She had a spontaneous vaginal delivery at 39 3/7 weeks and delivered a viable male infant weighing 3,035 grams with APGAR scores of 8 at 1 minute and 9 at 5 minutes.

## 3. Comment

Incarcerated uterus mimicking short cervix and placenta previa has not been reported in the past. Incarceration of the gravid uterus is a rare event with reported incidence of approximately one in 3000 pregnancies to one in 10000 [1–4]. Risk factors include retroverted uterus, endometriosis, pelvic adhesions, posterior wall leiomyomas, and deep sacral concavity and an overlying promontory [5–7]. Types of uterine incarceration include incarceration of an anteflexed gravid uterus and incarceration of a retroflexed uterus with or without sacculation [8]. Sacculation is the overstretching of the myometrium of the incarcerated uterus's anterior wall to expand the uterine cavity and accommodate the growing pregnancy [8]. Antepartum maternal symptoms include lower abdominal pain, constipation, urinary retention, and increased urinary frequency [1]. Intrapartum complications include labor dystocia, uterine rupture, retained placenta, and postpartum hemorrhage [9]. Complications may also include performing a cesarean delivery without the correct diagnosis, which may cause difficulties in identifying the bladder and the cervix, and, therefore, in opening the lower uterine segment. This may lead to bladder injuries, vaginal transection, or trans- or supracervical hysterectomy [1]. Examples of a complicated antepartum and intrapartum course include case reports by Gunn et al. [10] and Charova et al. [11] for two patients who underwent cesarean deliveries secondary to placenta previa [10, 11]. The hysterotomy incision was made through the posterior and anterior walls of the lower uterine segment and through the posterior wall of the fundus [11].

Attempts at correction of incarceration have been performed either manually, via laparotomy, or through colonoscopic release in the early second trimester of pregnancy [12]. Asymptomatic incarceration of the uterus has been reported until term [13]. High level of suspicion of incarceration of the uterus is very important to avoid major complications during the antepartum and the intrapartum stages [13].

Magnetic resonance imaging (MRI) has been suggested as a useful tool to diagnose incarcerated uterus [5]. A limitation of the management of our case is that we did not perform an MRI at around 21 weeks. Retrospectively, expectant management and spontaneous resolution of the incarceration proved to be the best option of management for our patient.

## 4. Conclusion

Our reported case emphasizes the importance of high suspicion for the diagnosis of incarcerated uterus in asymptomatic patients with difficult visualization of the cervix. The incarceration might present a fundal placenta as a low lying placenta, or as in our case, as a complete placenta previa. Key information for diagnosis includes unreachable or displaced cervix on physical and ultrasound examination and the cervix and lower uterine segment being located in the anterior wall of the uterus [5]. Magnetic resonance imaging (MRI) may be helpful in confirming the diagnosis [5]. Very close follow-up is recommended for the possibility of spontaneous resolution of the incarceration and the avoidance of procedures such as laparotomy or colonoscopy to restore the correct position of the uterus.

## Data Availability

This is a case report. Data that are pertinent to this case were presented in the manuscript.
